# Cardiac magnetic resonance in patients with pectus excavatum: impact of thoracic surgery on cardiac function - a follow- up-study

**DOI:** 10.1186/1532-429X-16-S1-P96

**Published:** 2014-01-16

**Authors:** Agnieszka Toepper, Susanne Poleichtner, Marcel Prothmann, Anja Zagrosek, Carsten Schwenke, Florian von Knobelsdorff, Klaus Schaarschmidt, Jeanette Schulz-Menger

**Affiliations:** 1 Working Group on Cardiovascular Magnetic Resonance, Experimental and Clinical Research Center, a joint cooperation between the Charite Medical Faculty and the Max-Delbrueck Center for Molecular Medicine, Berlin, Germany; 2 Cardiology and Nephrology, Helios Klinikum Berlin Buch, Berlin, Germany; 3 Cardiology, Charite Medical University Berlin, Berlin, Germany; 4 Helios Center of Pediatric and Adolescent Surgery, Helios Klinikum Berlin Buch, Berlin, Germany

## Background

Pectus excavatum (PE) as the most common anterior chest deformity is characterized by sternal depression with corresponding leftward displacement and rotation of the heart which is troublesome for assessment by ultrasound. Therefore CMR plays a growing role in preoperative evaluation. Indication for surgical correction is based on a diameter-based assessment of the thorax (Haller-Index, HI) and symptoms. Imaging data describing cardiac performance after surgery are rare. The aim of the study was to assess cardiac function during follow-up (FU).

## Methods

**Figure 1:**
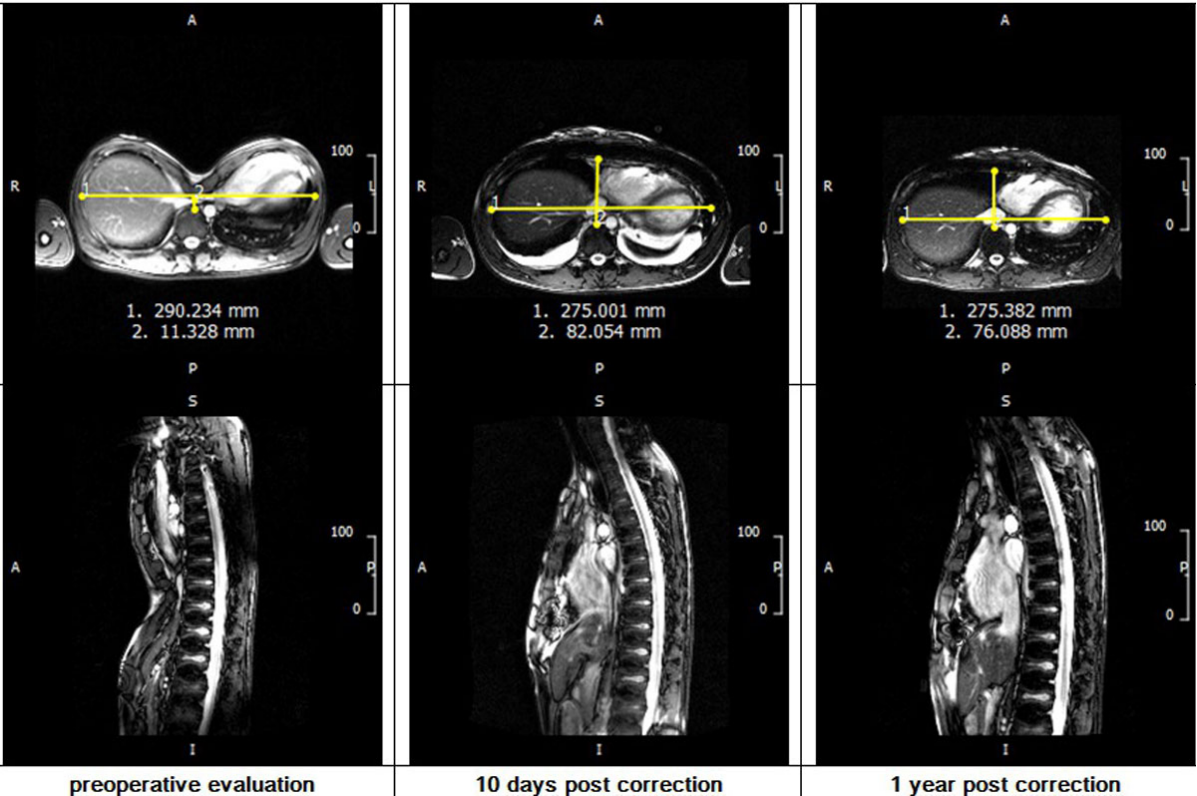


37 patients with PE (30 male, age 12-43 years) underwent CMR using 1.5T MRI-scanner (Avanto, Siemens Healthcare, Germany) prior to surgery and repeatedly postoperative (10 days, 3 months and 1 year). The highly experiences surgeons (more than 130 procedures/year) implanted a titan-bar (Nuss procedure) based on the usual clinical indication. CMR protocol included state of the art cine SSFP covering the left ventricle (LV) as short axis (TR 33.6 ms, TE 1.18 ms, FOV 292 × 360 mm, matrix 256 × 208, sth 7 mm without gap) and the right ventricle (RV) axial orientation (TR 34.56 ms, TE 1.21 ms, FOV 332 × 269 mm, matrix 256 × 208, sth 6 mm without gap). HI was measured on survey images (Figure 1). We quantified the enddiastolic and endsystolic volumes of LV and RV: calculated the ejection fractions (EF) and stroke volumes (SV) using CMR42 (circle cvi, Canada).

## Results

CMR images were evaluable despite implanted titanium bars. All patients completed one year FU. HI was significantly reduced after surgery (pre: 9.9 ± 5.7 vs. post: 2.8 ± 0.5, p < 0.001) indicating a successful procedure. Pleural effusions were detectable at day 10 in almost all (24 from 25 patients) pericardial effusion in 3 and it was resolved in all completely after 1 year. There were variable findings at different FU-time-points. The most positive change in RVEF was observed at 10 days FU followed by slight reduction on 3 months and further decrease at 1 year. Despite these findings the RVEF remained significantly improved at 1 year. Interestingly, RVEF was found to change significantly from baseline to 1 year FU (p < .0001), whereas LVEF did not (p = 0.0539). RVSV and LVSV were found to change significantly from baseline to 1 year follow-up (p = 0.0018 and 0.0008, respectively). Weak correlation was found between the delta (baseline/1 year) HI compared to RVEF.

## Conclusions

CMR is able to quantify LV and RV-morphology despite specific anatomy and/or implanted titanium bows in patients with pectus excavatum. Our results indicate that there is an improvement of stroke volumes and RVEF one year post correction.

## Funding

None.

